# Endodontic and Restorative Treatment Patterns of Pulpally Involved Immature Permanent Posterior Teeth

**DOI:** 10.1155/2018/2178535

**Published:** 2018-06-24

**Authors:** Ebtissam M. Al-Madi, Samar A. Al Saleh, Sundus M. Bukhary, Maha M. Al-Ghofaily

**Affiliations:** ^1^Department of Restorative Dentistry, College of Dentistry, King Saud University, P.O. Box 60169, Riyadh 11545, Saudi Arabia; ^2^Department of Clinical Dental Sciences, College of Dentistry, Princess Nourah bint AbdulRahman University, P.O. Box 84428, Riyadh 11671, Saudi Arabia; ^3^Department of Prosthetic Dental Sciences, College of Dentistry, King Saud University, P.O. Box 60169, Riyadh 11545, Saudi Arabia

## Abstract

**Objective:**

The study aims at investigating the treatment patterns for young permanent posterior teeth with pulp involvement.

**Materials and Methods:**

A random sample of 1793 dental records of patients aged 6–18 years old who had received dental treatment was investigated. 663 permanent posterior treated teeth had pulp involvement. Demographic and treatment data were gathered from patients' records.

**Results:**

Prevalence of young permanent teeth with pulp involvement was 36.9%. Treatments received significantly increased as patients' age increased (*P*=0.001). The first mandibular molar had the most pulp involvement among all teeth (43.89%). Temporary restoration was the most received restoration (59%). The most common pulpal diagnosis, leading to treatment, was irreversible pulpitis (43.04%). Only 19.8% of treated teeth received completed root canal treatment.

**Conclusion:**

There is a high percentage of children and adolescents with immature permanent posterior teeth with pulp involvement. Similarly, a variety of treatment patterns is present, with a small percentage of completed root canal treatment.

**Clinical Relevance:**

The study has identified the need to provide guidelines to provide high-quality root canal treatments for young permanent posterior teeth that have pulpal involvement. Only 21.8% of root canal treatments were completed, while 24% of teeth were extracted, and 59% of patients received temporary restorative treatments. This suggests that there might be several factors that might prevent completion of the dental treatment, such as patient preference, insurance coverage, or dentist capability. These factors and guidelines for patient care should be investigated and resolved.

## 1. Introduction

Dental caries is the most common infectious disease of childhood and adolescence and is rated the highest among dental problems [1, 2]. Caries prevalence has increased in the last three decades in Saudi Arabia [3]. If the cariogenic environment persists in the oral cavity, the newly erupted permanent teeth, specifically the first molar, may get decayed [4]. Young permanent teeth have wide dentinal tubules, a large pulp chamber, and high pulp horns [5], shortening the distance for decay to reach the pulp. If not treated early, infection of the dental pulp will eventually occur [6]. A high prevalence (35.8%) of young permanent teeth with pulp involvement was found in Saudi schoolchildren aged 6–18 years [7].

Treatment of pulpal infection in young permanent teeth in children and adolescence presents a unique challenge to dental clinicians [8]. Although there are many treatment options, evidence-based treatments are limited. Vital pulp techniques are associated with poor clinical outcomes, in the case of symptoms referring to irreversible pulpitis, such as unprovoked pain [9]. In addition, when the infectious process cannot be arrested by treatment methods to preserve vitality, such as protective liner, apexogenesis, indirect pulp treatment, direct pulp cap, and partial pulpotomy [10], then nonvital treatment options should be attempted, such as pulpectomy or apexification [11]. Extraction should only be considered if bony support cannot be regained, inadequate tooth structure remains for a restoration, or excessive pathologic root resorption exists [10, 12, 13]. If the tooth is preserved, it is imperative that the overlying intermediate and/or final restoration must be tight-sealed to decrease bacterial leakage from the restoration-dentin interface [14–18]. Root canal systems are morphologically complex, which makes them difficult to clean and fill. Therefore, root canal filling does not always prevent coronal bacterial contamination. This type of restoration poses a dilemma, especially in severely decayed permanent teeth, as the instability of occlusion at an early age presents a changing environment for a permanent restoration [19]. Therefore, in many instances, the decision is deferred, and consequences of delayed treatment increase the likelihood of failure.

This study aims to analyze the current treatment patterns for young permanent teeth with pulpal involvement (deep carious lesions and/or root canal treatment) in patients aged 6–18 years old in multiple centers in Riyadh, Saudi Arabia.

## 2. Methods and Materials

The Institutional Review Board (IRB) of the College of Dentistry, King Saud University, has deemed this study as exempt from IRB approval, due to the minimum risk to participants. In addition, written consents were embedded in the medical records of both institutes, and these have been approved by the IRB.

A retrospective evaluation of a random sample of 1793 dental records of male and female patients aged 6–18 years old who had received dental treatment at two different institutes (dental college clinics and military hospital) was performed. Dental records that indicated deep carious lesions and/or root canal treatment in young permanent posterior teeth were further analyzed.

Demographic data (patient file number, gender, and date of birth), treatment data (tooth number, pulpal diagnosis, and treatment provided), date of pulp extirpation and complete root canal treatment (if performed), and type of restoration placed were recorded. The periapical radiograph was examined to evaluate presence of any periapical lesions.

Prior to the analysis, data were anonymized and de-identified, and data were analyzed by using the Statistical Package for the Social Sciences (SPSS; Version #16). Frequency distribution was used for the descriptive analysis, and the chi-square test was used for statistical association between the variables. The significance level was set at 0.05.

## 3. Results

Six hundred sixty-three young permanent posterior teeth with pulpal involvement (deep carious lesions and/or root canal treatment) were identified and analyzed. The prevalence of immature permanent posterior teeth with pulpal involvement was 36.9%.

**Figure 1:**
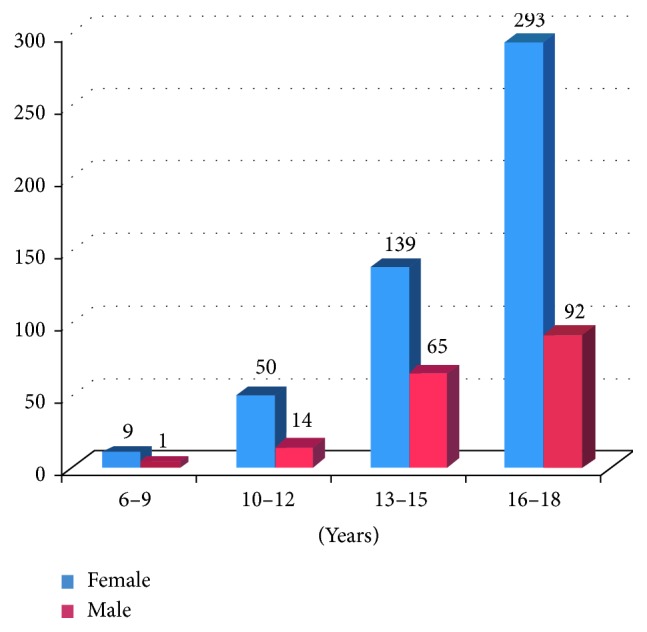
Distribution of young permanent teeth with pulpal involvement according to patients' age and gender.

The number of teeth with pulpal involvement significantly increased as patients' age increased (*P*=0.001). Significantly more molars had pulpal involvement than premolars (*P* < 0.05). The mandibular first molar was the most affected tooth (43.9%). The distribution of posterior teeth with pulpal involvement with regards to patient age is shown in Table 1. More than twice the number of female patients (73.3%) presented with pulpal involvement than males (26.7%). The distribution of young permanent teeth with pulpal involvement per patient age and gender is shown in Figure 1.

The most common pulpal diagnosis leading to treatment was irreversible pulpitis (43%) followed by pulp necrosis (33.27%) and reversible pulpitis (12.84%), while only 10.85% were diagnosed as vital pulp. The most common treatment was pulp extirpation (35.6%), while the least was apexification (0.2%). All teeth with vital diagnosis and 97% of teeth with reversible pulpitis were treated by vital pulp therapy. On the other hand, within teeth diagnosed with irreversible pulpitis, 70% had pulp extirpation performed, 25.6% had completed root canal treatment, and 0.1% were extracted. Among teeth with necrotic pulps, 34.7% had cleaning and shaping only, 32.6% had completed root canal treatment, while 31.5% were extracted. Distribution of pulpal diagnosis per treatment rendered is displayed in Table 2. All types of rendered treatments increased significantly as patients' age increased (*P*=0.001). The distribution of the treatment for teeth with pulpal involvement per patient age is shown in Table 3.

Although no radiographs were taken for 41.9% of the cases, in those which radiographs were taken, there was a periapical lesion associated with 32.7% of the teeth.

Overall, most of the teeth with pulpal involvement (59%) received temporary restorations (37.1% IRM and 21.9% Cavit), and only 17% received permanent restorations (9.4% GIC, 4% amalgam, 2.1% crowns, and 1.5% composite), while 24% of the involved teeth were extracted.

## 4. Discussion

Prevalence of young permanent posterior teeth with pulp involvement was found to be very high (36.9%) in the sample group. This is in agreement with a previous investigation that showed comparable results of 35.8% prevalence [7]. On the contrary, it contrasts with reports from American and European countries, where prevalence is much lower [20–23].

A significantly higher number of females had pulpal involvement in young permanent posterior teeth (74%) compared to males (26%). Tooth eruption occurs earlier in girls than boys; therefore, caries exposure and subsequently pulpal involvement could occur earlier [24]. Females have also been reported to visit the dentist more frequently than males [25]. Females might also be more able to vocalize their complaints about their teeth to their parents [7].

The number of young permanent posterior teeth with pulpal involvement and their treatment significantly increased as age increased. The largest age group with posterior teeth with pulpal involvement was the 16–18-year-old group. This is likely due to the cumulative nature of caries and pulpal involvement [26]. The most treatment provided was pulp extirpation (35.6%), followed by extraction (24%), despite the negative consequences to extraction. Although extraction might be a viable solution for teeth with irreversible pulpitis or necrosis in younger children, this is not a suitable solution for older adolescents [27]. Optimal treatment would involve complete endodontic therapy, followed by core buildup and a cusp protecting indirect restoration [28]. In this instance, only 1.5% of the involved cases in 6–9-year-olds were extracted, while an alarming 58.1% of the involved cases were extracted in the 16–18-year-old group. This warrants a review of the knowledge of guidelines for pediatric dental therapy among dentists in the area, as well as awareness among patients.

There were significantly more molars involved than premolars. The first mandibular molar was more prevalent within all age groups. The first molar has been quoted as the most caries-prone tooth in permanent dentition, probably due to its early exposure to the oral environment and its morphological features being pitted and fissured, inducing plaque and caries formation [29]. The most restoration used was temporary restoration, regardless of treatment rendered (59%). Most likely, as most treatment consisted of incomplete root canal therapy, the restoration of choice would be a temporary one. Another explanation would be that many clinicians would be unable to decide on a final treatment plan for the patient, as the final prognosis and outcome was unclear to them. It is difficult to attempt to perform final and permanent restorations in a young age group, since the lack of occlusal stability might provide a restorative dilemma. On the other hand, glass ionomer cements (GIC) with or without an IRM base have been suggested as a favorable long-term seal. IRM and GIC over a Cavit base over a one-month period have been suggested to provide a significantly superior seal against penetration of *S. mutans* when compared to Cavit alone [30].

Although most cases of irreversible pulpitis and necrosis (76.3%) had root canal treatment initiated, only a small number (21.8%) of these were completed. This could be explained by lack of ability of clinicians to commit to the completion of RCT, as the restorative choices are unclear.

Teeth with vital pulps or reversible pulpitis were treated by vital pulp therapy, while teeth with irreversible pulpitis were treated by pulp extirpation in most cases. Only a quarter of cases had a full root canal treatment completed. Within the group of teeth with necrotic pulps, an equal distribution of initiated RCT, complete RCT, and extractions were performed. Further investigations into the reasons behind the differences in treatment of cases of necrosis versus irreversible pulpitis need to be investigated.

Although many of the teeth proved to have periapical involvement, many had no documentation of X-rays to prove periapical involvement. There might be more periapical involvement in these teeth. This would warrant further treatment to eliminate disease progress by RCT completion and adequate restoration [31].

Overall, within the limitations of this study, results show a disturbingly high percentage of children and adolescence with pulpal involvement of young permanent teeth. Most of these teeth have partial pulpal treatment, with temporary restorations. Further investigations into clinicians' opinions regarding treatment options are currently being investigated to recognize the reasons behind this large variation in treatment patterns. Further prognostic studies are needed to establish the clear guidelines to the most advantageous restorative treatment options after endodontic therapy for this population.

## 5. Conclusion

Prevalence of young permanent posterior teeth with pulp involvement was found to be very high (36.9%) in Saudi Arabia. More females had young permanent posterior teeth with pulpal involvement (74%) compared to males (26%). The largest age group with immature permanent posterior teeth with pulp involvement was the 16–18-year-old group. The first mandibular molar was the most commonly affected tooth. The most treatment provided was pulp extirpation (35.6%), followed by extraction (24%). The most common restoration used was temporary restoration (59%), and although cases of irreversible pulpitis and necrosis (76.3%) had root canal treatment initiated, only a small number (21.8%) of these were completed. Further investigations and prognostic studies are needed to evaluate the reasons and prognosis of these treatment patterns.

## Figures and Tables

**Table 1:** Distribution of posterior permanent teeth with pulpal involvement treated teeth according to patients' age.

Age group	Max1PM	Max2PM	Mand1PM	Mand2PM	Max1M	Max2M	Mand1M	Mand2M	Total
6–9	0	0	0	0	5	0	5	0	10
10–12	1	4	0	1	15	2	40	1	64
13–15	13	6	2	3	61	8	99	12	204
16–18	26	27	4	23	97	34	147	27	385
Total	40	37	6	27	178	44	291	40	663
Total (%)	6.0%	5.6%	0.9%	4.1%	26.8%	6.6%	43.9%	6.0%	100%

Max1PM : maxillary first premolar; Max2PM : maxillary second premolar; Mand1PM : mandibular first premolar; Mand2PM : mandibular second premolar; Max1M : maxillary first molar; Max2M : maxillary second molar; Mand1M : mandibular first molar; Mand2M : mandibular second molar.

**Table 2:** Distribution of pulpal diagnosis according to treatment rendered.

	Extraction	Pulp extirpation	Vital pulp therapy	Root canal treatment	Apexification	Total
Vital	0	0	60	0	0	60 (10.8%)
Reversible	0	1	69	1	0	71 (12.8%)
Irreversible	4	167	6	61	0	238 (43%)
Necrosis	58	64	1	60	1	184 (33.3%)
Total	62 (11.2%)	232 (42%)	136 (24.6%)	122 (22%)	1 (0%)	553 (100%)

Vital : vital pulp; reversible : reversible pulpitis; irreversible : irreversible pulpitis: necrosis : necrotic.

**Table 3:** Distribution of treatment according to patients' age.

Age group	Extraction	Pulp extirpation	Vital pulp therapy	Root canal treatment	Apexification	Total (%)
6–9	3	1	5	0	1	10 (1.5%)
10–12	23	18	14	9	0	64 (9.7%)
13–15	36	78	46	44	0	204 (30.8%)
16–18	97	139	71	78	0	385 (58.1%)
Total	159	236	136	131	1	663
Total (%)	24%	35.6%	20.5%	19.8%	0.2%	100%

## Data Availability

The data used to support the findings of this study are available from the corresponding author upon request.
