# Corrigendum to “Endodontic and Restorative Treatment Patterns of Pulpally Involved Immature Permanent Posterior Teeth”

**DOI:** 10.1155/2020/9016926

**Published:** 2020-11-01

**Authors:** Ebtissam M. Al-Madi, Samar A. Al Saleh, Sundus M. Bukhary, Maha M. Al-Ghofaily

**Affiliations:** ^1^Department of Restorative Dental Sciences, College of Dentistry, King Saud University, P.O. Box 60169, Riyadh, Saudi Arabia; ^2^Department of Prosthetic Dental Sciences, College of Dentistry, King Saud University, P.O. Box 60169, Riyadh, Saudi Arabia

In the article titled “Endodontic and Restorative Treatment Patterns of Pulpally Involved Immature Permanent Posterior Teeth” [1], the affiliation [3] should be changed from “Department of Prosthetic Dental Sciences, College of Dentistry, King Saud University, P.O. Box 60169, Riyadh 11545, Saudi Arabia,” to the correct affiliation “Department of Restorative Dental Sciences, College of Dentistry, King Saud University, P.O. Box 60169, Riyadh 11545, Saudi Arabia.”

The corrected list of affiliations is shown in the authors' information above.
